# Evaluation of sealants to mitigate the release of per- and polyfluoroalkyl substances (PFAS) from AFFF-impacted concrete: Characterization and forecasting

**DOI:** 10.1016/j.wroa.2023.100195

**Published:** 2023-08-09

**Authors:** Phong H.N. Vo, Trent A. Key, Tu Hoang Le, Jeffrey T. McDonough, Scott Porman, Stephanie Fiorenza, Hong T.M. Nguyen, Vinh T.N. Dao, Jochen F. Mueller, Phong K. Thai

**Affiliations:** a Queensland Alliance for Environmental Health Sciences (QAEHS), The University of Queensland, Queensland, 4102, Australia; b Climate Change Cluster, Faculty of Science, University of Technology Sydney, 15 Broadway, Ultimo, NSW 2007, Australia; c ExxonMobil Environmental and Property Solutions Company, Spring, TX 77389, USA; d ExxonMobil Biomedical Sciences Inc., Spring, TX 77389, USA; e Nong Lam University Ho Chi Minh city, Ho Chi Minh City, Vietnam; f ERM, Denver, CO 80202, USA; g Mobil Oil Australia, Melbourne, VIC 3008, Australia; h Arcadis North America, Houston, TX 77042, USA; i School of Civil Engineering, The University of Queensland, Queensland, 4102, Australia

**Keywords:** Concrete, PFAS, Runoff modeling, Surface sealant, AFFF

## Abstract

Per- and polyfluoroalkyl substances (PFAS) within concrete pads impacted by historical firefighting training using aqueous film-forming foam (AFFF) may be potential secondary sources of PFAS due to surficial leaching. This study aimed to (i) characterize the effectiveness of two commercially available sealants (Product A and Product B) in mitigating leaching of five PFAS (e.g., PFOS, PFOA, PFHxS, PFHxA, 6:2 FTS) from concrete surfaces at the laboratory-scale, and (ii) develop a model to forecast cumulative leaching of the same five PFAS over 20 years from sealed and unsealed concrete surfaces. Laboratory trials demonstrated that both sealants reduced the surficial leaching of the five PFAS studied, and Product B demonstrated a comparatively greater reduction in surface leaching than Product A as measured against unsealed controls. The cumulative PFOS leaching from an unsealed concrete surface is estimated by the model to be about 400 mg/m^2^ over 20 years and reached asymptotic conditions after 15 years. In contrast, the model output suggests asymptotic conditions were not achieved within the modeled time of 20 years after sealing with Product A and 85% of PFOS was predicted to have leached (∼340 mg/m^2^). Negligible leaching of PFOS after sealing with Product B was observed ( < 5 × 10^−9^ mg/m^2^). Results from modeled rainfall scenarios suggest PFAS leachability is reduced from sealed versus unsealed AFFF-impacted concrete surfaces.

## Highlights

• Product B (organic-based sealant) is more effective than product A (inorganic-based sealant).
• PFOS cumulative mass loss of unseal pad reaches to steady state after 15 years (∼400 mg/m^2^).
• 85% of PFOS mass has been leached from the product A-sealed pad after 20 years (340 mg/m^2^).
• PFOS cumulative mass loss from the product B-sealed concrete pad is negligible (5 × 10^−9^ mg/m^2^).

## Graphical abstract

**Figure:**
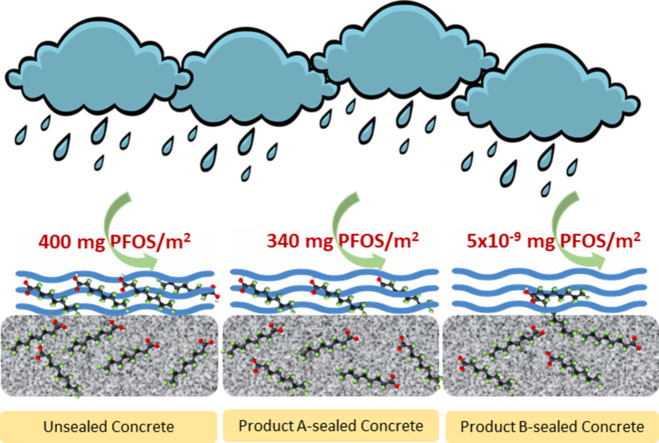

## Introduction

The use of aqueous film-forming foams (AFFF) containing per- and polyfluoroalkyl substances (PFAS) at some military bases, airports, and industrial facilities for firefighting and training purposes has impacted hardscape surfaces (i.e., firefighting training area concrete pads) (Douglas et al., 2023). These impacted hardscape surfaces may be a potential secondary source of PFAS to runoff due to surficial leaching (Thai et al., 2022). To potentially reduce the leaching of PFAS from the surfaces of AFFF-impacted hardscape, one pragmatic management and mitigation strategy to be considered is the use of sealants (Wang et al., 2020). Due to the current limited understanding, sealants should be evaluated through both short-term (laboratory-scale and/or field-scale) and long-term (field-scale and/or modeling) efforts to better evaluate the effectiveness of managing and mitigating surficial leaching of PFAS from AFFF-impacted hardscape surfaces.

A current limitation with respect to PFAS leaching from hardscape is that long-term loss of PFAS under dynamic conditions (i.e., rainfall) has not been fully considered, despite surficial leaching of PFAS during a few runoff events being evaluated (Thai et al., 2022). Hence, it is of value to forecast surficial leaching of PFAS from hardscape under dynamic conditions to understand potential inherent risks and mitigation strategies. Models may be employed to forecast surficial leaching of PFAS from concrete surfaces and improve the current understanding of potential long-term loss of PFAS under dynamic conditions based on parameters obtained from laboratory-based experiments.

In this study, short-term laboratory-scale experiments were conducted to assess the effectiveness of sealants to reduce surficial leaching of PFAS into runoff during simulated rainfall events. Further, a deterministic model was developed, evaluated, and applied to forecast surficial leaching of PFAS from sealed and unsealed AFFF-impacted concrete pads over a 20-year period.

## Results and discussion

### Release of PFAS from the sealed concrete cores

**Fig. 1:**
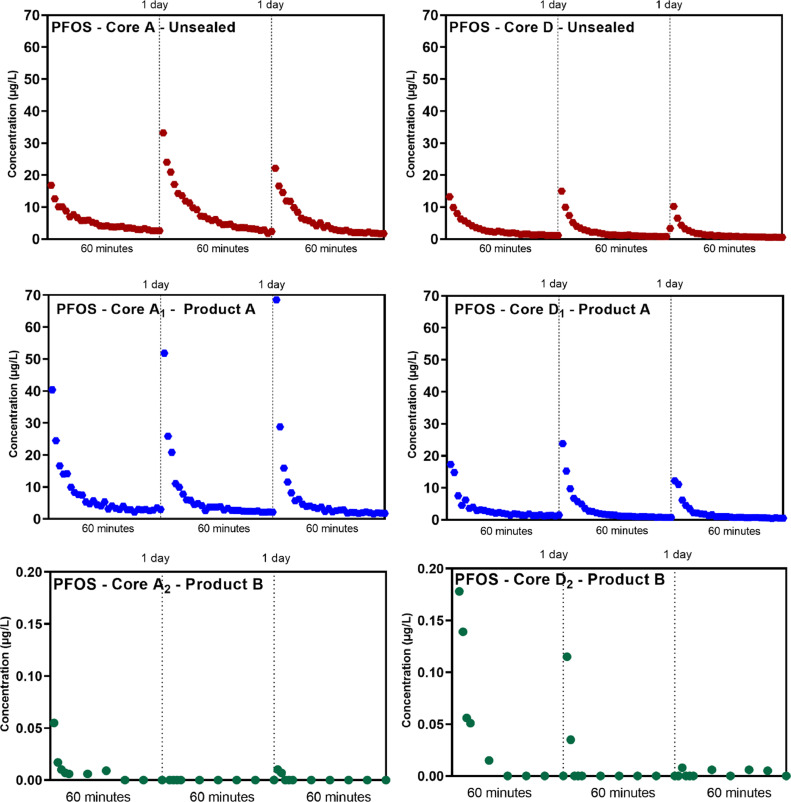
Profile of PFOS in runoff water from three rainfall simulations upon unsealed (reprinted from Thai et al. (2022)) and sealed concrete cores (with Product A and Product B).

The PFAS leaching results in water samples collected from concrete cores during the rainfall simulations are shown in Fig. 1 and Fig. S1–S4. Leaching profiles of all cores are subject to type A leaching suggesting a good match with the literature (Luo et al., 2013). Type A leaching is typically applicable for surfactant chemicals, in this case PFAS, with a rapid dissipation followed by asymptotic conditions. There is 3-fold difference (*p* < 0.01) between the leaching results from Product A-sealed cores and those of unsealed cores reported by Thai et al. (2022), noting initial PFAS mass concentrations differences in all the cores (Fig. S5) (*p* < 0.01). Further, there are substantially higher PFAS concentrations in leachate from the unsealed cores than Product B-sealed cores ( > 100 times). The maximum concentration of PFOS in runoff water from Product B-sealed cores was < 0.2 µg/L. There was an apparent decrease in the maximum PFOS concentration after each rainfall event that eventually reduced to below the limit of detection. PFAS leaching from Product B-sealed cores was significantly reduced (*p* < 0.01) with respect to unsealed controls under the experimental conditions tested. Other PFAS (e.g., perfluorohexanoic acid (PFHxA), perfluorooctanoic acid (PFOA), perfluorohexanesulfonic acid (PFHxS), and 6:2 fluorotelomer sulfonate (6:2 FTS) demonstrated similar leaching trends with respect to unsealed and sealed cores.

Given different mechanisms of leaching mitigation between Product A (e.g., a catalyzed crystallization within the concrete matrix) and Product B (e.g., an organic waterproof layer external to the concrete matrix), and the results summarized in Fig. 1, moisture wicking may be supported as previously described in Thai et al. (2022). Further, these sorption test data and differences in sealant mechanisms provide evidence to suggest the repeated surficial leaching of PFAS in successive rainfall simulations in unsealed and Product A-sealed concrete may be due to a variety of potential transport mechanisms (e.g., aggregation, adsorption, blocking) governed by the transmission of water inside the concrete matrix (Masoodi and Pillai, 2012). It is worth noting that sorption of PFAS at air-water interface in the microporous structure of concrete is one critical mechanism needing further investigation.

PFAS impacts associated with the historical AFFF use can represent decades of different types of AFFF and application methods (García et al., 2019). Correspondingly, previous investigations of the subject concrete pad sourcing the tested cores suggests a variable lateral and vertical extent of PFAS impacts (Baduel et al., 2015; Vo et al., 2023; Williams et al., 2023). For example, PFAS mass concentrations within cores collected near the middle of the concrete pad and the drainage area were approximately 2000-fold higher than cores collected from the outer extents of the pad. In addition, the modeled PFAS in this study represent a subset of the many potential PFAS that could be expected from historical AFFF use. Therefore, the nature of the PFAS mass concentration within the concrete is a critical factor in modeling PFAS leaching from the concrete.

The rate of water absorption was evaluated for sealed and unsealed cores. The organic sealant (Product B) reduced the rate of water absorption into the AFFF-impacted concrete matrix, which would otherwise facilitate surficial leaching and subsequent runoff of PFAS. Sorption tests performed following ASTM C1585 found initial and secondary rates of absorption were both 0.0 × 10^−4^ mm/√s for a Product B-sealed core compared to 1.7 × 10^−3^ mm/√s and 4.7 × 10^−4^ mm/√s of initial and secondary absorption rates for an unsealed core, respectively (Table S3). Conversely, the inorganic sealant (Product A) did not reduce the rate of absorption of water into AFFF-impacted concrete matrix. Product A facilitates a crystalline-based chemical reaction within the porosity of the concrete, attempting to stabilize PFAS. Initial and secondary rates of absorption were 2.8 × 10^−3^ mm/√s and 9.2 × 10^−4^ mm/√s, respectively, on a Product A-sealed core using sorption tests (ASTM C1585) (Table S3).

### Model development and evaluation

During calibration, the model used in this study generates a runoff hydrograph close to observed data (Fig. S6). Runoff (*h*_runoff_) started immediately at the beginning of the modeled rainfall scenario because the concrete reached saturation after 2–4 min and the infiltration (*h*_infiltration_) rate was low. Evaporation (*h*_evaporation_) and logging (*h*_logging_) had no impact on the runoff (*h*_runoff_) during the simulated rainfall event (60 min). Seemingly the rainfall intensity of 1 mm/min used in the experiments was sufficient to achieve constant runoff and negate the potential "wicking" effect attributable to evaporation (Masoodi and Pillai, 2012).

The values of EK_des_, estimated based on the data of the first rainfall simulation, and its performance indices (i.e., NSE and CV(RMSE)) are shown in Table 1. For PFOS, the estimated EK_des_ of the unsealed core (53 L/kg) is two times less than the Product A-sealed core (120 L/kg), suggesting that the former can leach ∼2-fold more PFAS than the latter over the experimental period. Further, Product B-sealed cores showed a minor leaching and EK_des_ decreased after each rainfall event. EK_des_ of the Product B-sealed core could not be comparably modeled to the unsealed and Product A-sealed cores as many data points of the leaching profiles were at or below the LODs for the five PFAS. However, for comparative purposes, EK_des_ for PFOS was estimated for the first two rainfall simulations of Product B-sealed cores to be 32,000 L/kg (NSE = 0.94, CV(RMSE)=  0.014) and 110,000 L/kg (NSE=  0.76, CV(RMSE)=  0.014), respectively.

**Table 1:** Values of estimated EK_des_ and calibrated and evaluated model performance indices.

Analyte	Core	EK_des_ (L/kg)	NSE	CV(RMSE)
PFOS	Unsealed core D	53	0.90	0.47
			0.88*	1.1*
	Product A-sealed core D_1_	120	0.91	1.1
			0.87*	1.7*
PFOA	Unsealed core D	27	0.93	0.054
			0.83*	0.066*
	Product A-sealed core D_1_	100	0.89	0.039
			0.72*	0.076*
PFHxS	Unsealed core D	7.8	0.95	0.34
			0.73*	0.74*
	Product A-sealed core D_1_	74	0.86	0.89
			0.73*	1.3*
PFHxA	Unsealed core D	11	0.95	0.34
			0.77*	0.30*
	Product A-sealed core D_1_	190	0.90	0.22
			0.74*	0.51*
6:2 FTS	Unsealed core D	5.8	0.97	0.61
			0.64*	2.1*
	Product A-sealed core D_1_	68	0.55	0.29
			0.76*	0.30*

Without asterisk (*): calibration, with asterisk (*): evaluation.

Note: EK_des_ for Product B-sealed cores were not included because of limited estimated values due to concentrations below the LOD.

After fitting the model to the experimental results, the model was evaluated for validity using data from replicated rainfall simulations from the same concrete cores. Most NSE values of the evaluation are > 0.70, which indicate reasonable predictive performance of the model (N. Moriasi et al., 2007). The comparatively small CV(RMSE) values also indicate reasonable predictive performance (N. Moriasi et al., 2007). This suggests that the model reasonably represents the empirical conditions and can therefore be used to predict the resultant PFAS leachability after sealing the concrete cores in this study with Product A and Product B relative to unsealed controls.

### Forecasting PFAS loss due to rainfall runoff in 20 years from a concrete pad

**Fig. 2:**
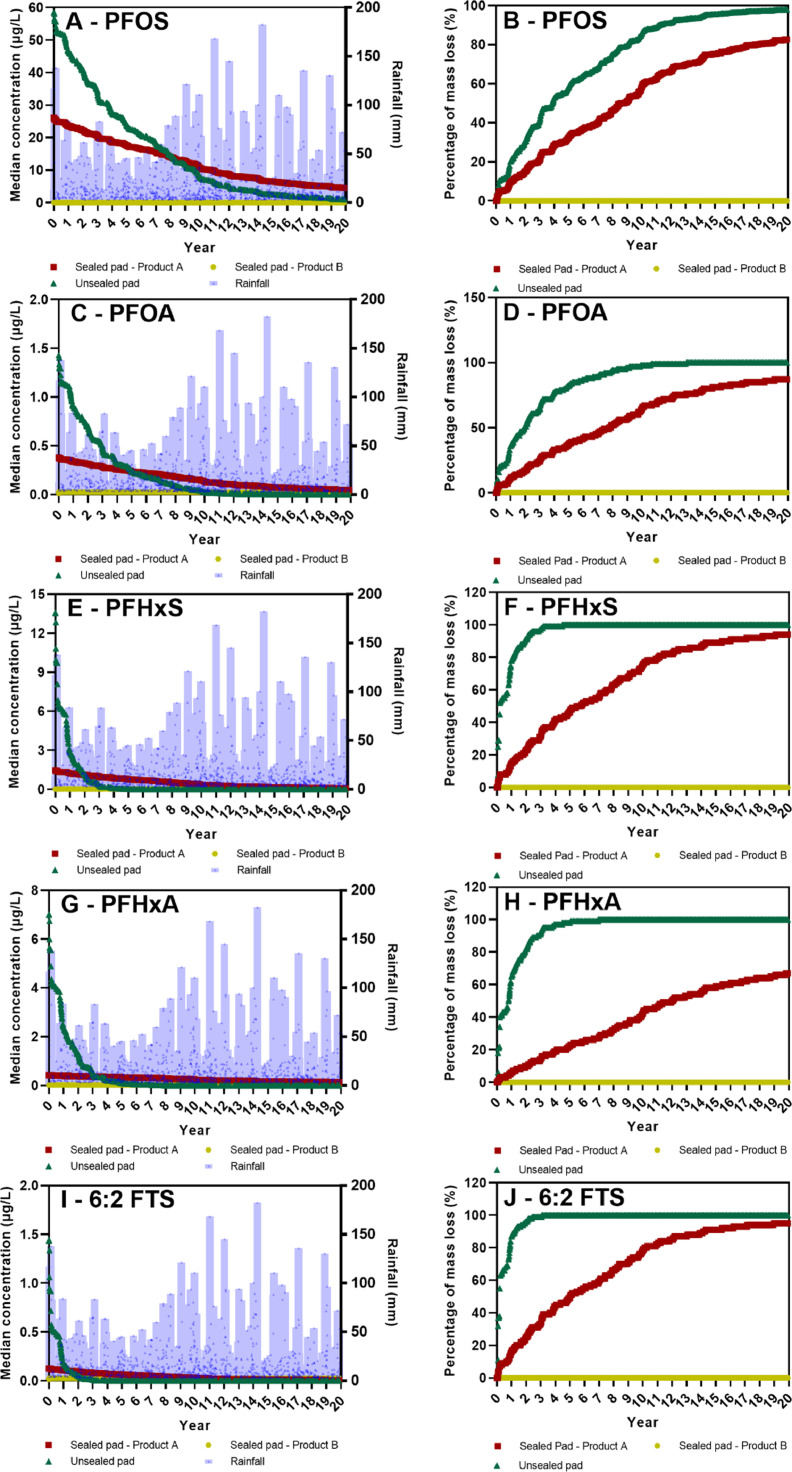
Forecasted five PFAS median leaching concentration of each rainfall event for 20 years (Panel A, C, E, G, I) and cumulative mass loss of five PFAS from sealed and unsealed concrete pads (Panel B, D, F, H, J). The symbol used to represent rainfall depth is a purple dot within a purple bar to allow for simultaneous graphical representation of both the discrete rainfall depth data (purple dots) and the maximum rainfall depth for a given year (top of purple bar).

The model was used to forecast PFAS runoff from the concrete pad characterized in Baduel et al. (2015) under three scenarios: (1) the unsealed pad, (2) the pad sealed by Product A, and (3) the pad sealed by Product B. Forecasting over 20 years was performed using historical rainfall data. The forecasted median concentrations and mass loss of the studied PFAS are shown in Fig. 2 and Fig. S7, respectively. The forecasted median PFOS leaching concentration from the unsealed and Product A-sealed pad appears to decrease over the modeled 20 years, starting at approximately 60 and 25 µg/L and ending at approximately <1 µg/L and 4 µg/L, respectively.

It is noteworthy that the forecasted median PFOS leaching concentration from the Product A-sealed pad was lower than the unsealed pad for years 1–8. For other PFAS, the median leaching concentration in the Product A-sealed pad is less than the unsealed pad for years 2–5. This suggests that the use of the Product A sealant on AFFF-impacted concrete surfaces may slow the leaching of PFAS to runoff compared to unsealed concrete surfaces. A potential explanation for this is that Product A sealant formed a non-soluble crystalline precipitate within the concrete matrix likely reducing the ability for entrained water (and dissolved PFAS within the entrained water) to leach from the concrete.

The PFOS cumulative mass loss of the unsealed pad appears to reach asymptotic conditions after 15 years (∼400 mg/m^2^), indicating near-complete (97%) PFOS leaching from the top 0.5 cm. Approximately 85% of PFOS mass from the Product A-sealed pad is forecasted to have been leached over the 20-year modeled rainfall scenario (∼340 mg/m^2^), and asymptotic conditions are not reached until after the study timeframe. The unsealed pad and Product A-sealed pad appear to leach approximately 50% of the PFOS after approximately 4 and 9 years, respectively. These are 2.5 to 6 times shorter than the 25 years estimated by Baduel et al. (2015). The PFOS cumulative mass loss from the Product B-sealed concrete pad appears to be negligible, and the total PFOS mass loss modeled based on data from the first two rainfall simulations (Fig. 1) is less than 0.01 µg (equivalent to <5 × 10^−9^ mg/m^2^). The leaching results from the other 4 PFAS studied closely resemble that of PFOS for both Product A- and Product B-sealed cores.

### Limitations of forecast modeling

Despite the findings summarized herein, the model remains limited in the following aspects. Firstly, this model does not incorporate various potential fate and transport mechanisms that may be occurring, such as the advection of potential moisture wicking and the potential for PFAS supramolecular structures and/or bilayer formation controlled dissolution (Krafft and Riess, 2015; Lombardo et al., 2015). Owing to extremely strong intramolecular forces, amphiphilic PFAS tend to self-assemble into various supramolecular structures (Krafft and Riess, 2009). The formation of these structures can control the rate of dissolution of the PFAS and therefore the rate of leaching. Sorption and transport of colloidal particles may also influence leaching, either decreasing PFAS leaching through slow advection and diffusion into the concrete matrix or increasing PFAS leaching due to detachment of PFAS adhered to colloids (Borthakur et al., 2021a). Another limitation regarding the leaching process is that the EK_des_ was derived empirically from experimental data using only one rainfall intensity (1 mm/min over 60 min) that may vary if the rainfall intensity changes.

The durability of the sealant to endure physical and chemical stresses on the sealed surface and natural concrete weathering also likely challenge the confidence of the modeling (Borthakur et al., 2021b; Douglas et al., 2023). Routine vehicle trafficking and the use of chemical agents (e.g., lubricant, fuel) are suggested to cause deterioration of sealants over time. Sealant degradation over time is the subject of multiple separate and ongoing studies, which is especially pertinent to evaluate applicability of the organic-based sealant (Product B) to surfaces intended to be used for fire training where temperatures may routinely exceed product melt and/or flash point(s). Further, one study found diminished degradation may be observed using microcapsule technology to improve the self-healing property of the sealant even at subzero temperatures (down to −20 °C) (Sun et al., 2020). Natural concrete weathering due to environmental factors such as elevated temperature and rainfall can reduce the binder adhesion of the sealant to the concrete (Hung et al., 2017). Small imperfections in the sealed surface may absorb water, facilitating the diffusion of moisture into the binder, which ultimately undermines the adhesion of the sealant. The diffusion of moisture into the sealant is reported to increase with increasing temperature (Hung et al., 2017).

Further, the mechanics of actual rainfall were not included in study design, and incorporating the kinetic energy, droplet size, and runoff velocity of water in future efforts could be more representative of real-world conditions (Mikelonis et al., 2021). The selection of concrete depth for conducting modeling is another area of improvement. Though PFAS were found to be concentrated in surficial and upper portions of concrete matrix in these samples, the vertical distribution may differ based on site-specific conditions as well as individual PFAS constituents (Vo et al., 2023; Williams et al., 2023). For example, PFHxS was concentrated at 1–6 cm while the peak PFOS concentration was observed from 0–4 cm (Vo et al., 2023). More studies are needed to better understand the movement of PFAS within the concrete matrix.

While the modeling approach has merit, the variability of concrete chemistry/characteristics and erosion has not been fully represented. It might result in differences in absorption, diffusion, and leaching of PFAS. Significant changes in leaching of organic chemicals were reported for stamped concrete (an imprinted and textured concrete), which may be attributable to the increasing surface roughness and physical trapping (Jiang et al., 2012). Whether this is also true for PFAS leaching remains questionable and worthy of additional investigation. Further, this study focused on five anionic PFAS. The leaching of cationic and zwitterionic PFAS and their relevance to the effectiveness of the two sealants tested are subjected to investigation in future study.

## Conclusion

The reduction of PFAS leaching from AFFF-impacted concrete surfaces due to the application of two different sealants suggests that sealants may serve as a mitigation strategy to manage PFAS leaching from concrete depending on site-specific conditions. Commercial concrete sealants have been used in many applications, and this study evaluates their ability to mitigate PFAS leaching. The use of concrete sealants to reduce PFAS leaching must also achieve site-specific requirements for the intended purpose of the concrete infrastructure (e.g., slip resistance, UV resistance, thermal resistance, etc.). While Product B significantly reduced PFAS leachability from a concrete surface in comparison to an unsealed concrete surface, its organic composition may be incompatible with surfaces used for future fire training. Future work will evaluate the field-scale durability of multiple PFAS-relevant sealants to better understand the options available to mitigate potential PFAS leaching from concrete surfaces. The deterministic model described herein was used to forecast a 20-year comparison of the tested experimental conditions to support evaluation of the potential benefits of managing PFAS leaching from concrete with two sealants. Further, these results suggest that use of concrete sealants may be a viable management strategy to mitigate PFAS leaching from AFFF-impacted concrete.

## Materials and methods

### Concrete cores, sealants, and rainfall simulations

Six cylindrical concrete cores (*d* = 120 mm; *h* = 10 cm) were collected from an AFFF-impacted concrete pad using a core drill (Thai et al., 2022). After drilling, two cores were sealed with each selected sealant (Product A and Product B) and stored in individual plastic bags to prevent cross-contamination. Two additional cores were left unsealed to serve as controls. Product A and Product B are sealant products used for concrete repair and water proofing, respectively. Product A is an inorganic-based sealant with function as a catalyst to form non-soluble crystalline structures in the inner pores of the concrete. Product B is an organic-based bitumen-like sealant that can prevent water absorption into concrete. Rate of sorption tests of the concrete cores were performed following ASTM C1585 (Standard Test Methods for Measurement of Rate of Absorption of Water by Hydraulic-Cement Concretes) (ASTM International, 2020).

The PFAS mass concentration within the concrete was determined by drilling 4 to 5 holes around the circumference of the concrete core at different depths (i.e., core sidewall drilling) to develop a profile (0.5 cm, 1 cm, 2 cm, 3 cm, 4 cm, 6 cm, 8 cm, and 10 cm). Concrete dust samples from the drilling were composited, extracted, and analyzed as described previously (Thai et al., 2022). Five PFAS were studied: PFHxA, PFOA, PFHxS, PFOS, and 6:2 FTS. The selected PFAS are the most prevalent in AFFF-impacted concrete reported in previous studies (Baduel et al., 2015; Thai et al., 2022).

Rainfall simulation experiments were conducted to contain both wetting and drying periods, as described previously (Thai et al., 2022). Rainfall was applied at the rate of 1 mm/min for a duration of 60 min and runoff was collected at 2 min interval. The runoff samples from cores were first spiked with a mass labelled internal standard and analysed via direct injection. Due to the low PFAS concentrations, the runoff samples from cores sealed with Product B were concentrated 20-times by solid phase extraction (SPE) prior to analysis. More details about the chemical analysis and the QA/QC results can be found in the Supporting Information (SI). One way analysis of variance (ANOVA) was performed to test the significant difference of leaching results.

## Development of a model for forecasting PFAS leaching through rainfall runoff

### Model development

**Fig. 3:**
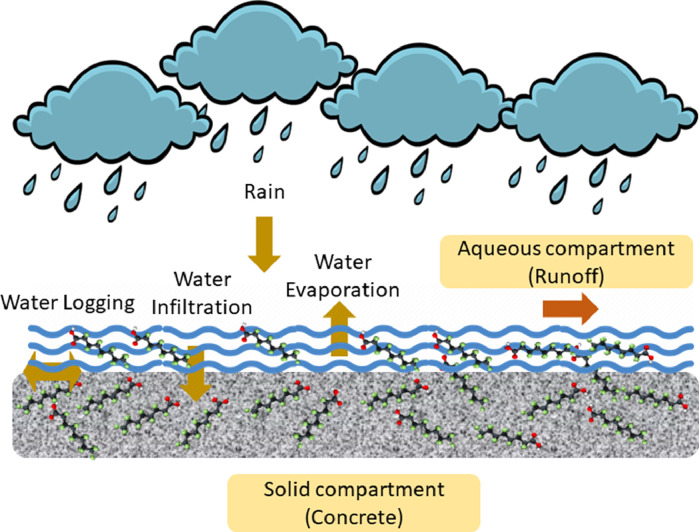
Conceptual diagram of main components of the model for leaching of PFAS from concrete (solid compartment) to runoff (aqueous compartment).

A deterministic model for forecasting surficial leaching of PFAS in rainfall runoff from an AFFF-impacted concrete pad was developed based on the principle of mass balance and mass transfer. This modeling approach has been validated in a model for estimating pesticide runoff (Phong et al., 2011). The model consists of three governing equations: the water balance on the concrete surface (Eq. (1)), the mass balance of PFAS in the water layer on the concrete surface (Eq. (2)), and the mass balance of PFAS within the porosity of the concrete (Eq. (3)). The main components of the model that impact PFAS leaching are shown in Fig. 3.

In a rainfall event, the water balance can be expressed by the following:

(1) $$h_{\text{runoff}}=h_{\text{rain}}-h_{\text{infiltration}}-h_{\text{evaporation}}-h_{\text{logging}}$$

where *h*_runoff_: runoff depth (mm), *h*_rain_: rainfall depth (mm), *h*_infiltration_: water infiltration depth into the concrete pad (mm), *h*_evaporation_: water evaporation depth in the local meteorology conditions (mm), *h*_logging_: water logging depth on the concrete surface (mm). Methods for estimating each component are presented in the SI.

Mass of PFAS lost to runoff (*M*_PFAS leaching_) is calculated as follows:

(2) $$M_{\text{PFAS}\;\text{leaching}}=h_{\text{runoff}}*S*\frac{C_{s}}{EK_{\text{des}}}\;$$

where *M*_PFAS leaching_: mass of PFAS leaching (µg), S: surface area of the leaching concrete (m^2^), C_s_: concentration of PFAS in concrete solid compartment (µg/kg), and effective K_des_ (EK_des_): where K_des_ represents a desorption coefficient, EK_des_ represents a lumped coefficient expressing the migration of PFAS from the solid compartment to the liquid compartment (L/kg). A lumped coefficient is intentionally specified to acknowledge that the mechanisms controlling PFAS leaching from concrete are currently not well understood. A more detailed description of estimating EK_des_ from experimental data can be found in the SI (Fig. S8).

The mass balance of PFAS associated with the surficial concrete is as follows:

(3) $$M_{\text{PFAS}\;\text{concrete}}={M^{o}}_{\text{PFAS}\;\text{concrete}}-M_{\text{PFAS}\;\text{leaching}}$$

where *M*^o^_PFAS concrete_: initial mass of PFAS in concrete before a rainfall event and *M*_PFAS concrete_: mass of PFAS remaining in concrete after a rainfall event (µg). For modeling purposes, only the top 0.5 cm of the concrete pad was considered, based on prior observations of PFAS mass primarily in surficial and upper portions of concrete matrix (Vo et al., 2023). Further, it was assumed that upward movement of PFAS within the concrete matrix through potential wicking processes would primarily occur within the top 0.5 cm; this model assumption was based upon 0.5 cm reported as the maximum pore-length in high-strength concrete (Vicente et al., 2018).

The model program was coded using Visual Basic for Applications incorporated in Microsoft Excel®. The model was developed to run in daily timestep. The concentrations and cumulative PFAS mass loss are calculated for each rainfall event.

### Model evaluation

The model was calibrated by manually tuning EK_des_ to define the range of near-optimal values. Subsequently, 1000 simulations of EK_des_ were performed within the defined range of EK_des_ determined via manual model tuning (Table 1). The optimal values of EK_des_ were chosen based on the best performance indices (i.e., Nash–Sutcliffe Efficiency [NSE] and Coefficient of Variation of the Root Mean Square Error [CV(RMSE)]) (Table 1).

The performance of the model is evaluated by two objective functions (i.e., NSE and CVRMSE) to evaluate model uncertainty, which are expressed as below:

(4) $$\text{NSE}\;=\;1-\;\frac{\sum_{t=1}^{T}{\left(C_{i}-C_{m}\right)}^{2}}{\sum_{t=1}^{T}{\left(C_{i}-\bar{C}\right)}^{2}}$$

(5) $$\text{CV}\left(\text{RMSE}\right)\;=\;\frac{100}{\bar{C}}\sqrt{\frac{\sum_{i=1}^{n}{\left(C_{m}-C_{i}\right)}^{2}}{n}}$$

where C_i_ and C_m_ are the observed and simulation concentration from the concrete cores, respectively, $\bar{C}$ is the average of the observed data, and n is the number of observations in the dataset.

### Model application

The model was applied to forecast cumulative mass loss of PFAS due to rainfall runoff from a concrete pad over 20 years. Concrete pad parameters, such as the surface area of the concrete and PFAS concentrations in concrete, were retrieved from Baduel et al. (2015). The rainfall data (daily rainfall depths) were retrieved from local meteorology station (Meteorology, 2022). Since the rainfall data are only available in form of cumulative daily depth, they were fed to the model as one rainfall event (*h*_rain_) for the days when rainfall occurred. The model was applied to forecast PFAS leaching in different scenarios: unsealed (control) and sealed (with Product A and Product B) concrete pads. The cumulative mass loss in 20 years is calculated by summing the mass loss of each rainfall event.

The input parameters for the model are shown in Table 2.

**Table 2:** Detail of the model input parameters.

Parameter	Unit	Description	Values
S	m^2^	Surface area of the concrete for model development	0.00635 (core) 518 (pad)
*h*_rain_	Mm	Rainfall depth	0.2–182.6
h	M	Depth of the concrete layer for modeling	0.005
ρ	kg/m^3^	Bulk density of the concrete	2400
t	Min	Modeled time of a rainfall event	0–60
T	Year	Modeled time to forecast PFAS mass loss accumulation	0–20
C_0_	µg/kg	Initial concentration of PFAS on the concrete surface at depth 0.5 cm	0–10,000
EK_des_	L/kg	Lumped coefficient	PFOS (50–120); PFOA (20–120); PFHxS (6–85); PFHxA (8–200); 6:2 FTS (5–80)

## CRediT authorship contribution statement

**Phong H.N. Vo:** Conceptualization, Methodology, Formal analysis, Investigation, Writing – original draft. **Trent A. Key:** Investigation, Formal analysis, Writing – review & editing. **Tu Hoang Le:** Methodology, Formal analysis, Investigation. **Jeffrey T. McDonough:** Methodology, Writing – review & editing. **Scott Porman:** Methodology, Writing – review & editing. **Stephanie Fiorenza:** Methodology, Writing – review & editing. **Hong T.M. Nguyen:** Methodology, Formal analysis. **Vinh T.N. Dao:** Writing – review & editing. **Jochen F. Mueller:** Methodology, Writing – review & editing. **Phong K. Thai:** Conceptualization, Methodology, Formal analysis, Investigation.

## Declaration of Competing Interest

The authors declare that they have no known competing financial interests or personal relationships that could have appeared to influence the work reported in this paper.

## Data Availability

Data will be made available on request.

## Appendix Supplementary materials

Image, application 1
