# Supplementary material for: Evaluation of sealants to mitigate the release of per- and polyfluoroalkyl substances (PFAS) from AFFF-impacted concrete: Characterization and forecasting
Source: Water Res X. 2023 Aug 9;20:100195. doi: 10.1016/j.wroa.2023.100195 (PMC10448196; doi:10.1016/j.wroa.2023.100195)
Supplement: Supplementary file 1 [file mmc1.docx]

**Evaluation of sealants to mitigate the release of per- and polyfluoroalkyl substances (PFAS) from AFFF-impacted concrete: characterization and forecasting**

Phong H. N. Vo^1,2 *^, [Trent A. Key](https://www.sciencedirect.com/science/article/pii/S266691102200003X#!)^3,4^, Tu Hoang Le^5^, [Jeffrey T. McDonough](https://www.sciencedirect.com/science/article/pii/S266691102200003X#!)^6^, [Scott Porman](https://www.sciencedirect.com/science/article/pii/S266691102200003X#!)^7^, Stephanie Fiorenza^8^, Hong T. M. Nguyen^1^, Vinh T. N. Dao^9^, [Jochen F. Mueller](https://www.sciencedirect.com/science/article/pii/S266691102200003X#!)^1^, [Phong K. Thai](https://www.sciencedirect.com/science/article/pii/S266691102200003X#!)^1^

^1^ Queensland Alliance for Environmental Health Sciences (QAEHS), The University of Queensland, Queensland, 4102, Australia

^2^ Climate Change Cluster, Faculty of Science, University of Technology Sydney, 15 Broadway, Ultimo, NSW 2007, Australia

^3^ ExxonMobil Environmental and Property Solutions Company, Spring, TX 77389, USA

^4^ ExxonMobil Biomedical Sciences Inc., Spring, TX 77389, USA

^5^ Nong Lam University Ho Chi Minh city, Ho Chi Minh City, Vietnam

^6^ ERM, Denver, CO 80202, USA

^7^ Mobil Oil Australia, Melbourne, VIC 3008, Australia

^8^ Arcadis North America, Houston, TX 77042, USA

^9^ School of Civil Engineering, The University of Queensland, Queensland, 4102, Australia

*Corresponding Author: phong.vo@uts.edu.au

**List of figures**

Fig. S1. Profile of PFOA in runoff water in three rainfall simulations upon unsealed.

Fig. S2. Profile of PFHxA in runoff water in three rainfall simulations upon unsealed.

Fig. S3. Profile of PFHxS in runoff water in three rainfall simulations upon unsealed.

Fig. S4. Profile of 6:2 FTS in runoff water in three rainfall simulations upon unsealed.

Fig. S5. PFAS profile on top surface (0.5 cm) of two unsealed* and four sealed concrete cores.

Fig. S6. Runoff hydrograph of a rainfall event.

Fig. S7. Mass loss (µg) in 20 years of PFOS, PFOA, PFHxA, PFHxS, 6:2 FTS.

Fig. S8. Conceptualization of EK_des_

**List of supporting information (SI)**

SI 1. Horton equation

SI 2. Kidd and Lowring equation

SI 3. Penman equation

SI 4. Studied PFAS and isotopically internal standards.

SI 5. Analysis and extraction of PFAS by LC-MS

SI 6. Quality assurance and quality control

SI 7. Assumption and estimation of EK_des_

SI 8. Rate of absorption into sealed and unsealed concrete

List of figures


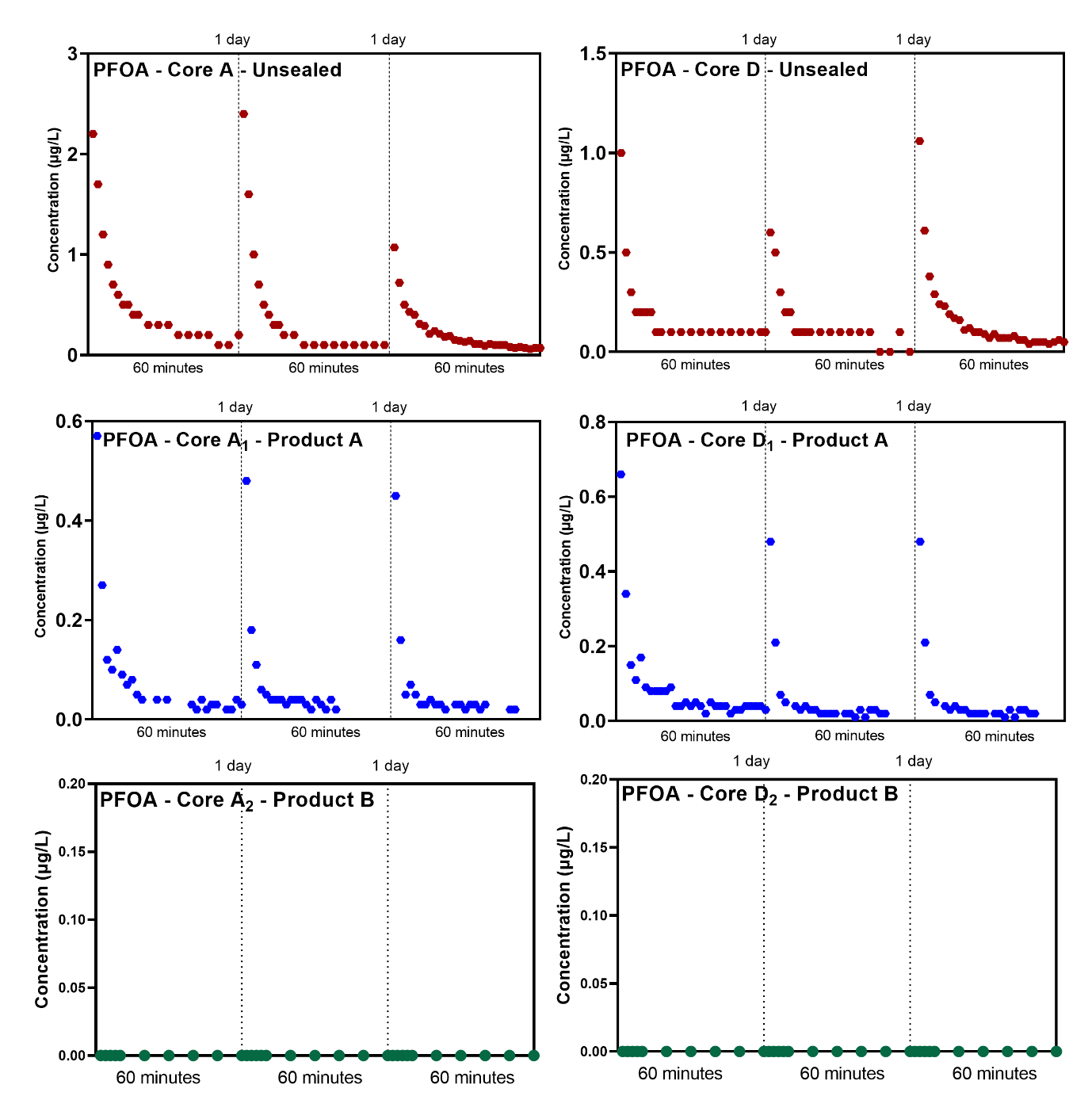


Fig. S1. Profile of PFOA in runoff water in three rainfall simulations upon unsealed (reprinted from Thai et al. (2022)) and sealed concrete cores (with Product A and Product B).


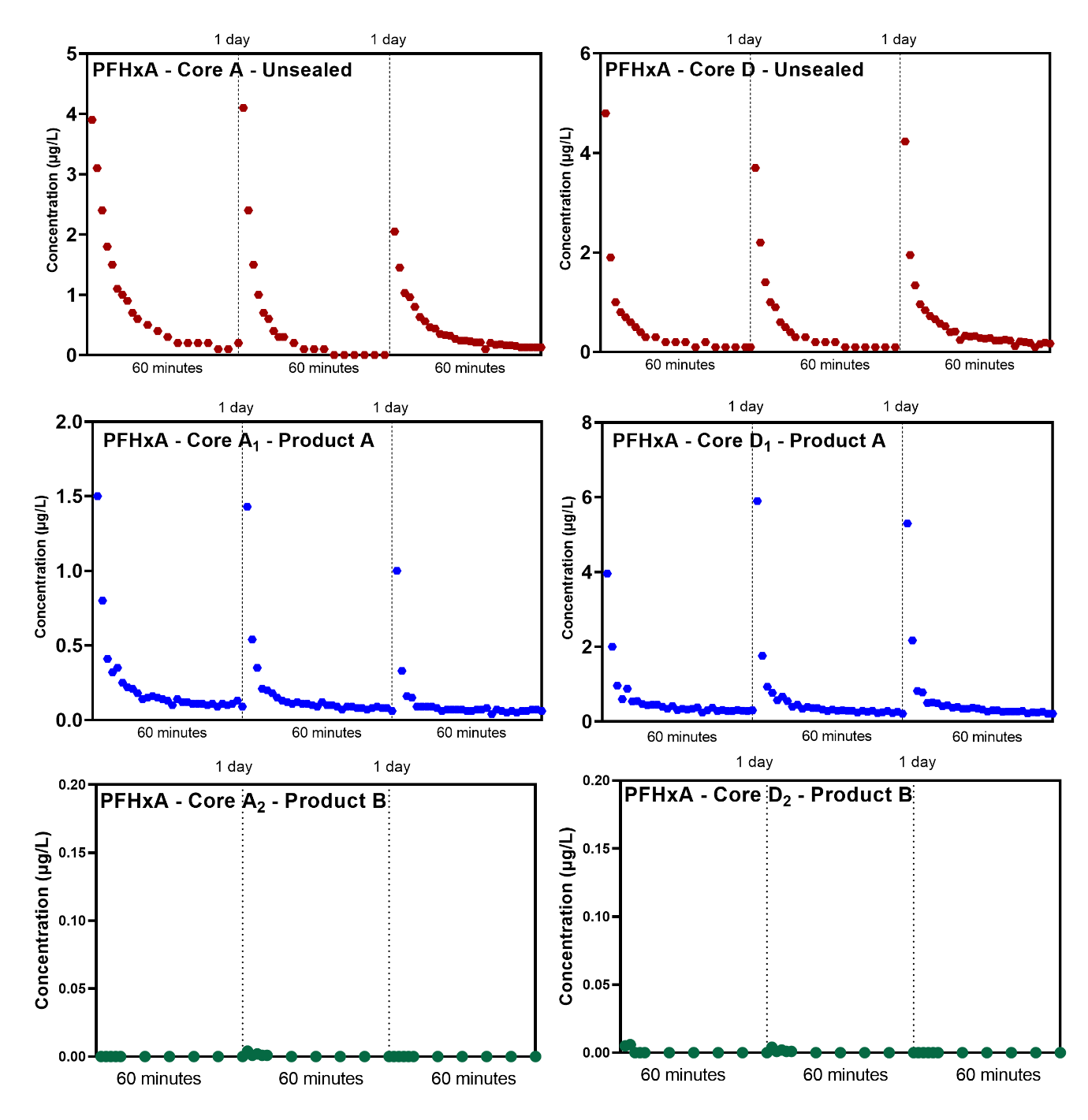


Fig. S2. Profile of PFHxA in runoff water in three rainfall simulations upon unsealed (reprinted from Thai et al. (2022)) and sealed concrete cores (with Product A and Product B).


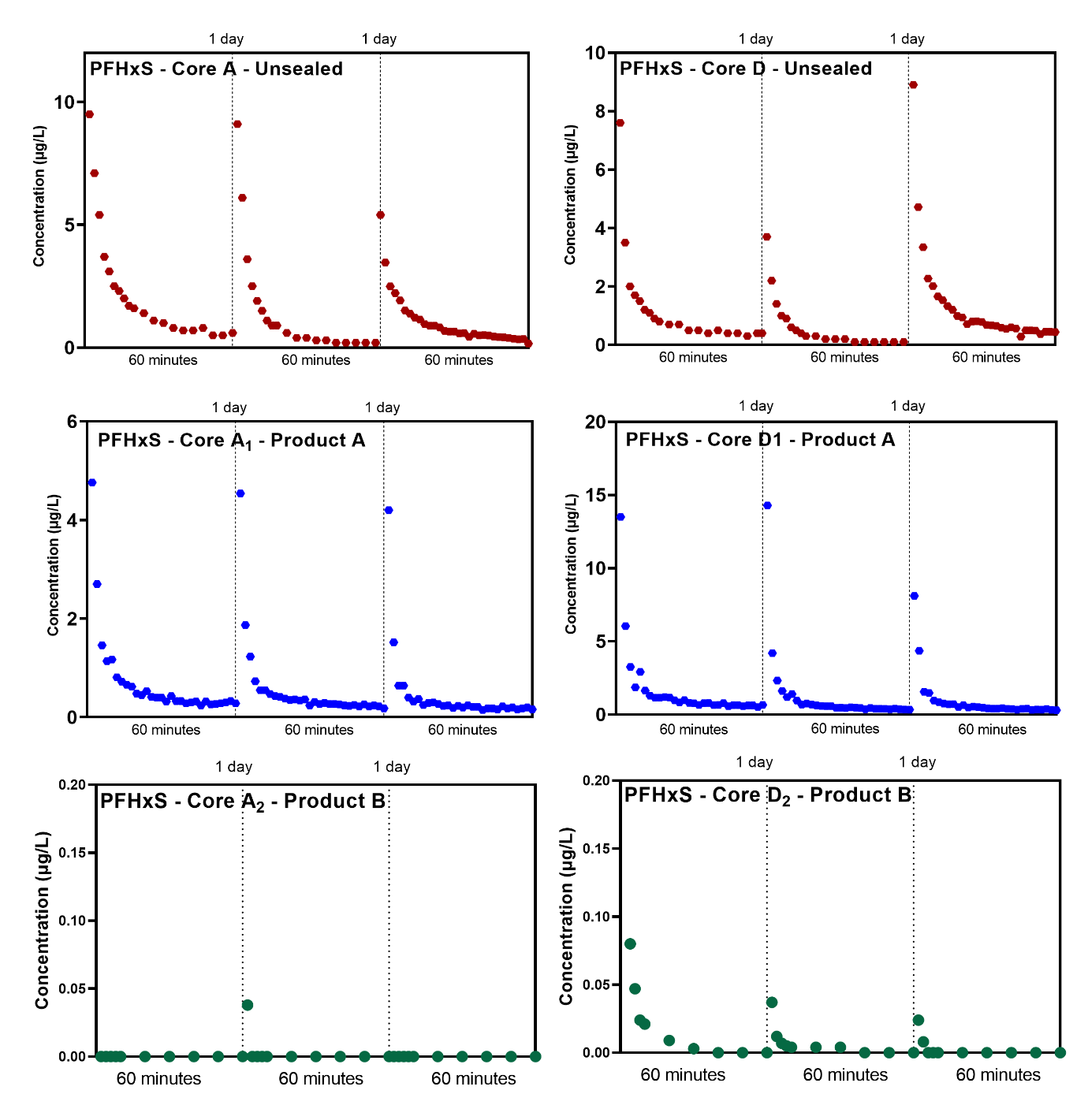


Fig. S3. Profile of PFHxS in runoff water in three rainfall simulations upon unsealed (reprinted from Thai et al. (2022)) and sealed concrete cores (with Product A and Product B).


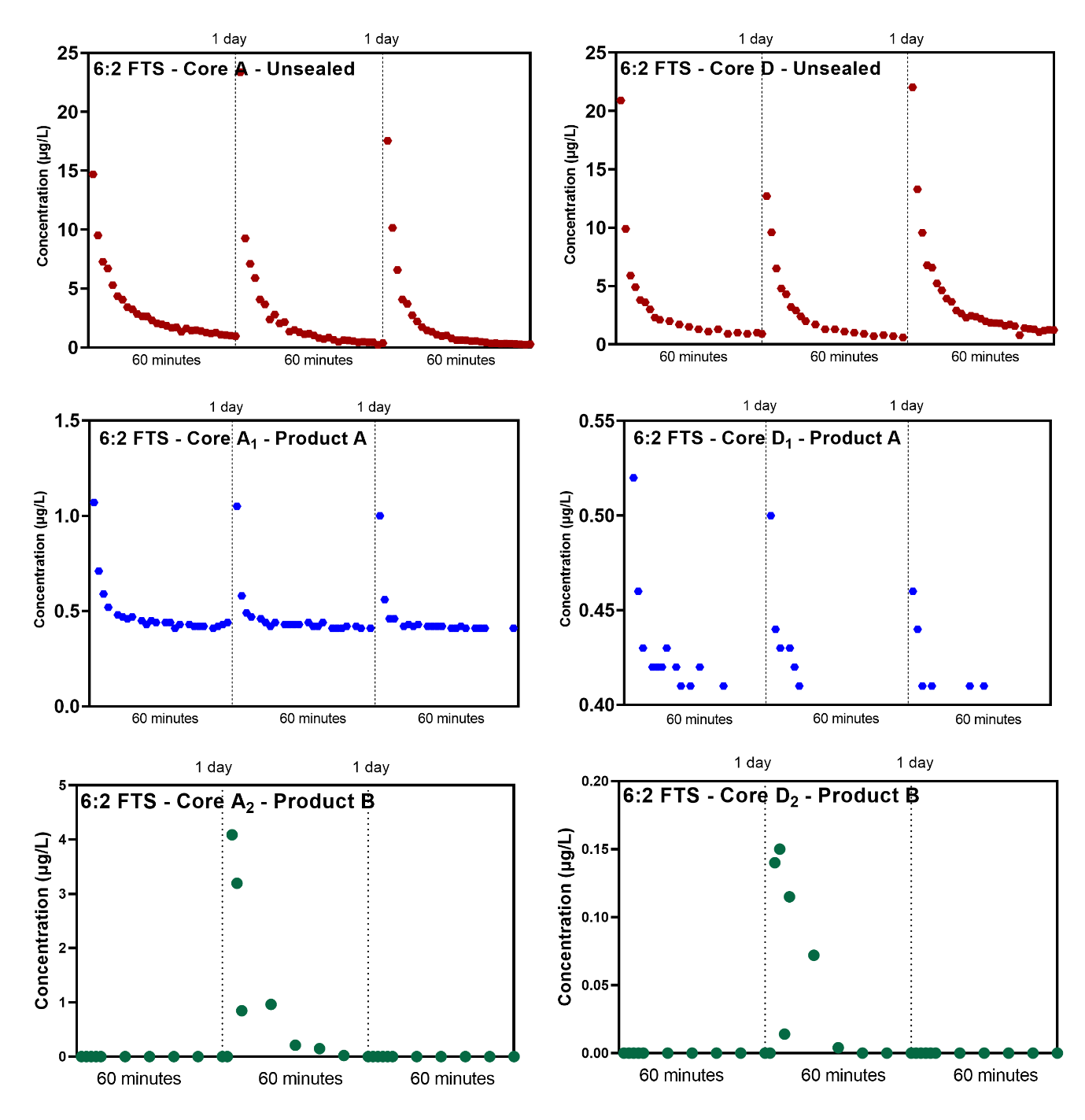


Fig. S4. Profile of 6:2 FTS in runoff water in three rainfall simulations upon unsealed (reprinted from Thai et al. (2022)) and sealed concrete cores (with Product A and Product B).


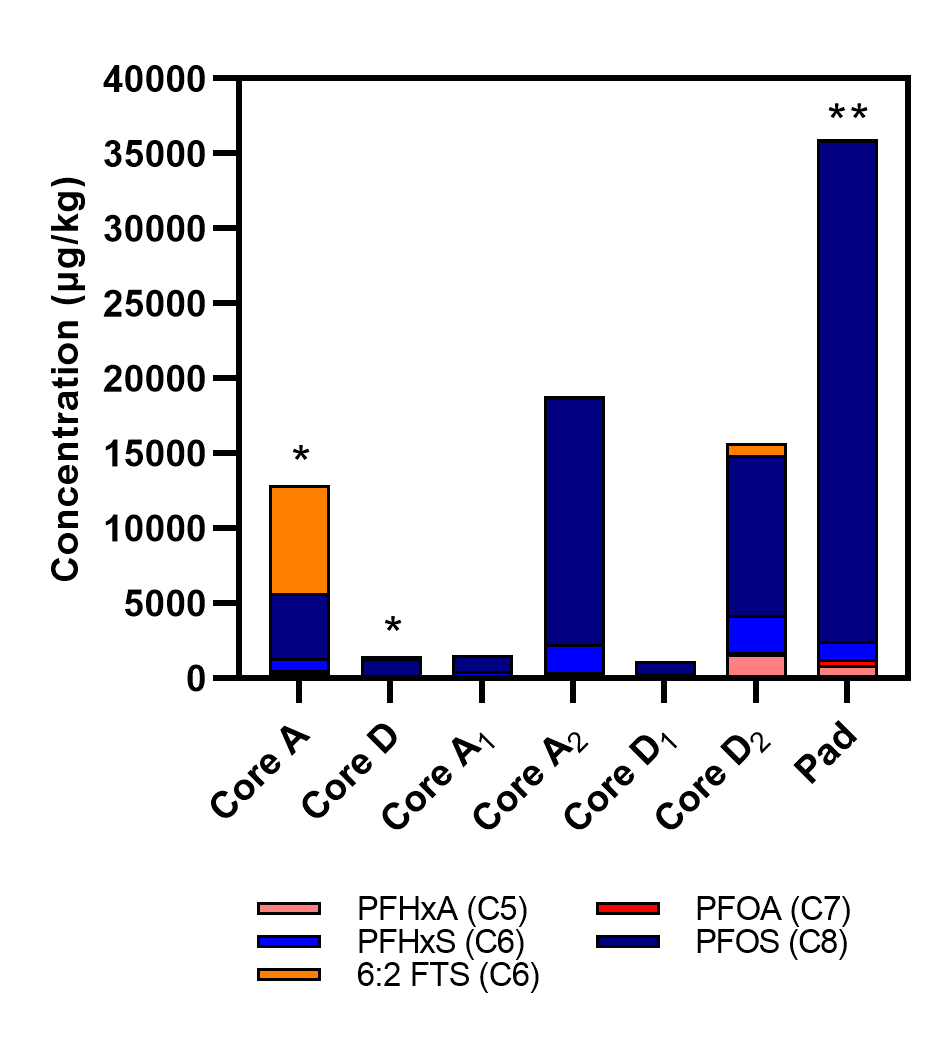


Fig. S5. PFAS profile on top surface (0.5 cm) of two unsealed* and four sealed concrete cores (two Product A-sealed cores [A_1_ and D_1_] and two Product B-sealed cores [A_2_ and D_2_]) and concrete pad**. The profile of two unsealed cores was retrieved from Thai et al. (2022), and concrete pad was retrieved from Baduel et al. (2015).


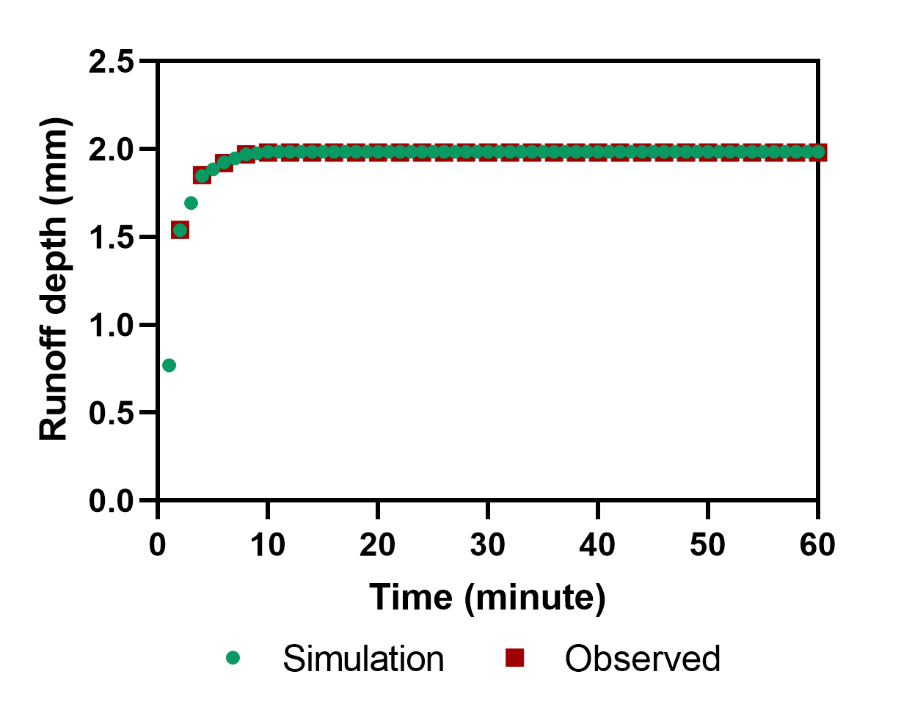


Fig. S6. Runoff hydrograph of a rainfall event


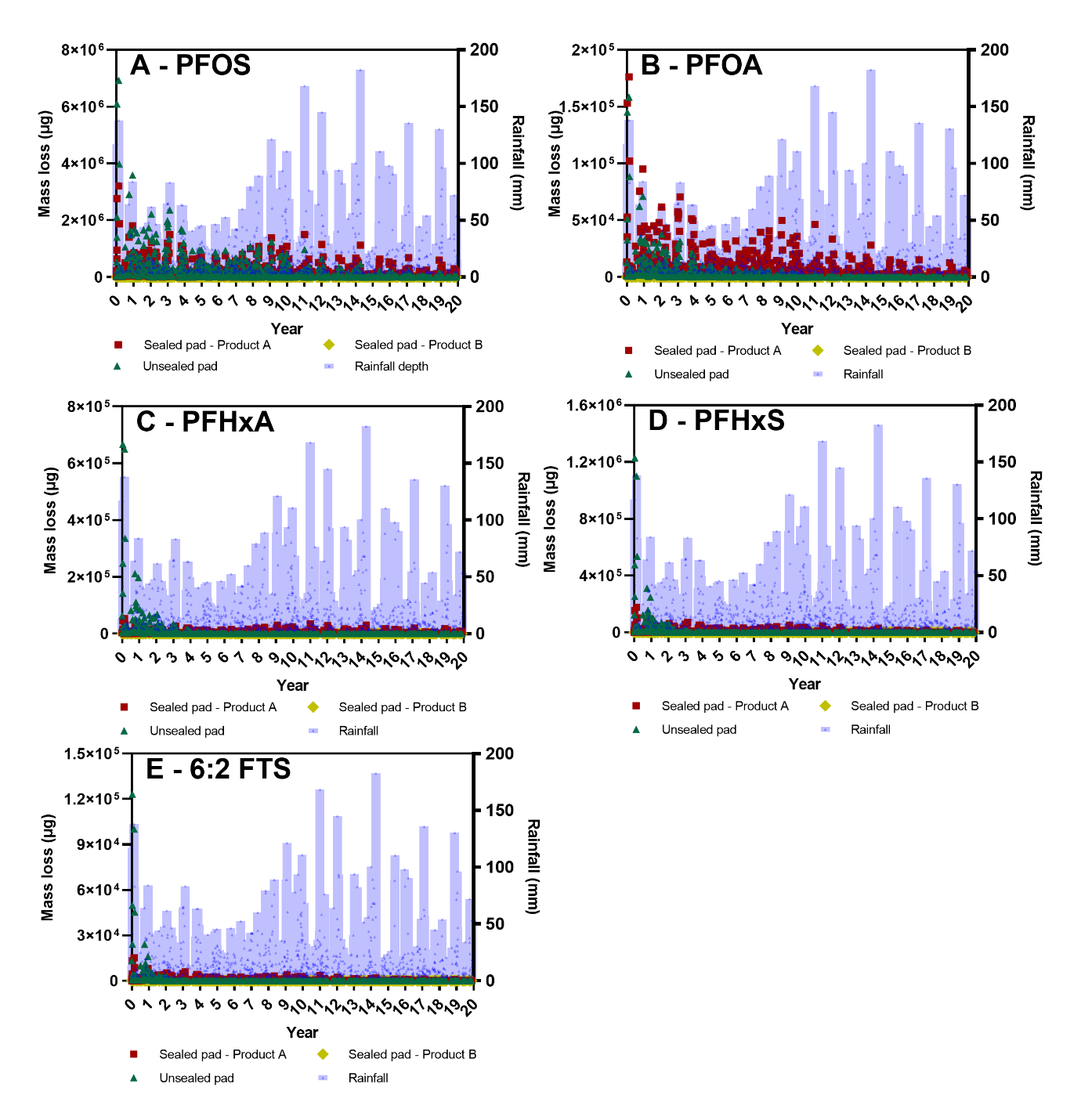


Fig. S7. Mass loss (µg) in 20 years of PFOS (Panel A), PFOA (Panel B), PFHxA (Panel C), PFHxS (Panel D), 6:2 FTS (Panel E).

**List of supporting information (SI)**

SI 1. Horton equation

The Horton equation is commonly used to represent infiltration (Butler et al., 2018):

f_t_ = f_c_ + (f_o_ – f_c_).e-^kt^

Where:

f_t_: infiltration rate at time t (mm/h)

f_c_: final (steady state) infiltration rate or capacity (mm/h)

f_o_: initial rate (mm/h)

k: decay constant (h^-1^)

SI 2. Kidd and Lowring equation

The Kidd and Lowring equation is used to represent the logging/depression of water on the concrete surface (Butler et al., 2018):

$$d=\frac{k_{1}}{\sqrt{s}}$$

Where:

k_1_: coefficient depending on surface type (0.07 for impervious surfaces and 0.28 for pervious surfaces) (mm)

s: ground slope

SI 3. Penman equation

The simplified Penman equation is used to estimate the evaporation rate from open water (Valiantzas, 2006). The evaporation rate is minor (<0.2 mm/d) which agrees with Butler et al. (2018).

E_Pen_≈0.047$R_{s}\sqrt{T+9.5}-2.4\left( \frac{R_{s}}{R_{a}} \right)^{2}+0.09(T+20)(1-\frac{RH}{100})$

SI 4. Studied PFAS and isotopically internal standards.

Total 5 PFAS were studied comprising of 2 PFCAs (Perfluorohexanoic acid (PFHxA), Perfluorooctanoic acid (PFOA)) and 2 PFSAs (Perfluoro hexanesulfonate (PFHxS), Perfluoro octanesulfonate (PFOS)) and 1 PFAAs precursor (1H,1H,2H,2H perfluorooctane sulfonate (6:2 FTS)) (Table S1). For internal standards, 5 mass labelled PFAS including 13C2-PFHxA, 18O_2_-PFHxS, 13C_2_-6:2 FTS, 13C_4_-PFOA, 13C_4_-PFOS were used for quantification (Table S1).

Table S1. PFAS classes and their acronyms in this study

| PFAS class/name | Number of perfluorinated carbon | Formula | Acronym | Mass (g/mol) | IS |
| --- | --- | --- | --- | --- | --- |
| Perfluoroalkyl carboxylates (PFCAs) C_n_F_2n+1_COO^-^ | 5 | C_6_F_11_O_2_- | PFHxA | 313.05 | 13C4-PFOA |
|  | 7 | C_8_F_15_O_2_- | PFOA | 413 | 13C4-PFOA |
| Perfluoroalkyl sulfonates (PFSAs) C_n_F_2n+1_SO_3_^-^ | 6 | C_6_F_13_O_3_S- | PFHxS | 399 | 18O2-PFHxS |
|  | 8 | C_8_F_17_O_3_S- | PFOS | 499 | 13C4-PFOS |
| X:2 Fluorotelomer sulfonates (FTSs) C_n_F_2n+1_CH_2_CH_2_SO_3_^-^ | 6 | C_8_H_4_F_13_O_3_S- | 6:2 FTS | 428.1 | 13C2-6:2FTS |

SI 5. Analysis and extraction of PFAS by LC-MS

The concrete samples (0.1 g) were extracted by 3 ml of ammonia methanol (2%) and 2 ml acetone after spiking 10 µL of mass labelled internal standard (0.2 ppm). The sample was vortexed, followed by sonication for 20 min and tumbled for 15 min. The sample was centrifuged at 4150 RCF for 15 min and the extract was aliquoted. The residual was extracted again using the same procedure. Two aliquots were mixed and loaded on an Envi-carb carbon cartridge (6 ml, 250 mg). The cartridge was rinsed with 1 ml ammonia methanol. The eluate was blown down to dry and reconstituted in 0.5 ml methanol/water (1:1 v/v). The aqueous surficial leaching (i.e., runoff samples) (0.5 ml) of Product A-sealed cores were spiked with mass labelled internal standard and analysed via direct injection. Due to the low level of PFAS presented, the runoff samples of Product B-sealed cores were condensed 20-times by solid phase extraction (SPE) prior to analysis.

The extract and runoff samples were analysed for PFAS using liquid chromatography coupled tandem mass spectrometry (LC-MS/MS 5500). The detail of instrumental analysis is provided in appendix S1. The QA/QC control was performed using a duplicate sample (per 10 samples) and native spiked samples (per 20 samples). The calibration set was run at the beginning and end of the batch. The QA/QC results were compatible to the standard of US DoD/DoE Consolidated Quality System Manual (version 5.3) for environmental laboratories (DoD and DoE, 2019)

SI 6. Quality assurance and quality control

The calibration standards were injected at the beginning and the end for instrumental drift. Duplication of samples were performed. The quality control sample was injected each ten samples. PFAS concentration was quantified by linear regression analysis of the calibration curve (R^2^ > 0.993). Limits of quantification (LOQ) were chosen at ten times the standard deviation of the lowest standard by eight injections. The detection limit (LOD) was set as three times of the standard deviation of the lowest concentration of the standard after 8 injections. The standard for signal-to-noise was set at 10. Recoveries of internal standard is provided in Table S2.

Table S2. Quality assurance and quality control

| PFAS | IS | LOD (**μg/L**) | LOQ (**μg/L**) | Internal standard (IS) recovery (%) (n=154) |
| --- | --- | --- | --- | --- |
| PFHxA (C5) | 13C4-PFOA | 0.04 | 0.14 | 94.9 ± 4.8*  76.1 ± 15.7** |
| PFOA (C7) | 13C4-PFOA | 0.03 | 0.1 | 88.9 ± 6.7*  75.2 ± 15.2** |
| PFHxS (C6) | 18O2-PFHxS | 0.03 | 0.1 | 77.6 ± 9.9*  69.5 ± 18.3** |
| PFOS (C8) | 13C4-PFOS | 0.05 | 0.17 | 108.9 ± 6.1*  62.6 ± 18.5** |
| 6:2 FTS (C6) | 13C2-6:2FTS | 0.03 | 0.1 | 81.5 ± 5.8*  95.6 ± 28.5** |

* Internal standard (IS) recoveries of rainfall samples direct injection.

** Internal standard (IS) recoveries of PFAS extraction from concrete.

SI 7. Assumption and estimation of EK_des_

To minimize the discrepancy of observed and simulation data, effective K_des_ (EK_des_) was calibrated using experimental data conducted in this study and retrieved from Thai et al. (2022). EK_des_ is differentiated from K_des_ because it is a lumped coefficient expressing the migration of PFAS from the solid compartment to the liquid compartment. It was hypothesized that EK_des_ in this model is comprised of surface bound PFAS, dissolved PFAS in concrete porosity, and self-assembled PFAS within and at the concrete surface, which results in both desorption and slow advection/diffusion from the concrete matrix (Fig. S8). The model was then validated using replicate experimental data of the same cores. The calibration and validation procedures comprise of the following steps. At first, a possible range and data distribution of K_des_ values were tuned manually. There is a lack of information relevant to the distribution of K_des_ values, thus we assumed a uniform distribution. Upon determining the range of K_des_, a Latin Hypercube sampling technique was used to randomly generate EK_des_ values for the simulation. To ensure a high accuracy of the model, 1000 simulations were performed, and accuracy of each simulation was evaluated using the objective functions (NSE and CV(RMSE)) defined in section 2.2.2. The derived value of EK_des_ with the highest NSE and CV(RMSE) was then used for model calibration and model application.


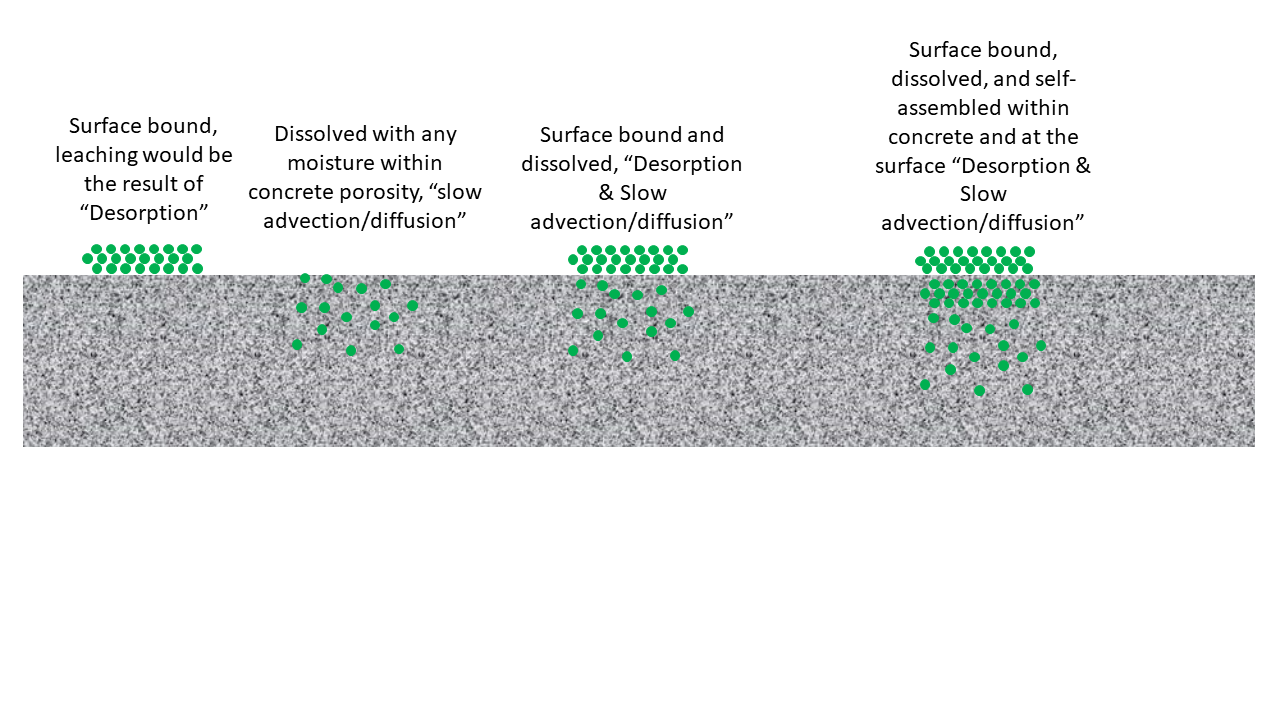


Fig. S8. Conceptualization of EK_des_

SI 8. Rate of absorption into sealed and unsealed concrete

Sorptivity tests were performed following ASTM C1585 on sealed (Product A and Product B) and unsealed concrete cores to measure initial and secondary rates of absorptions. Sorptivity tests were performed by Boral Construction Materials (NSW, Australia). The results are reported in Table S3 below.

Table S3. Rate of Absorption into Sealed and Unsealed Concrete

| Sample ID | Lab Sample No. | Date of Test (mm/dd/yy) | Diameter of the Sample (mm) | Rate of Absorption (mm/√s) | |
| --- | --- | --- | --- | --- | --- |
|  |  |  |  | Initial* | Secondary** |
| Core D_1_  (Product A-sealed) | 218974 | 04/06/19 | 119.1 | 2.8x10^-3^ | 9.2x10^-4^ |
| Core D_2_  (Product B-sealed) | 218975 | 04/06/19 | 119.0 | 0.0x10^-4^ | 0.0x10^-4^ |
| Core D  (Unsealed Control) | 218976 | 04/06/19 | 119.0 | 1.7x10^-3^ | 4.7x10^-4^ |

* Initial rate of absorption (mm/√s) calculated from 1 minutes up to 360 minutes.

** Secondary rate of absorption (mm/√s) calculated from 24 hours up to 192 hours.

**References**

Baduel, C., Paxman, C.J., Mueller, J.F., 2015. Perfluoroalkyl substances in a firefighting training ground (FTG), distribution and potential future release. *Journal of Hazardous Materials*, 296, 46-53. <https://doi.org/10.1016/j.jhazmat.2015.03.007>

Butler, D., Digman, C., Makropoulos, C., Davies, J.W. 2018. *Urban Drainage*. *4th ed*. CRC Press.

DoD, DoE. 2019. Department of Defense (DoD) Department of Energy (DOE) consolidated quality systems manual (QSM) for environmental laboratories.

Thai, P.K., McDonough, J.T., Key, T.A., Thompson, J., Prasad, P., Porman, S., Mueller, J.F., 2022. Release of perfluoroalkyl substances from AFFF-impacted concrete in a firefighting training ground (FTG) under repeated rainfall simulations. *Journal of Hazardous Materials Letters*, 3, 100050. <https://doi.org/10.1016/j.hazl.2022.100050>

Valiantzas, J.D., 2006. Simplified versions for the Penman evaporation equation using routine weather data. *Journal of Hydrology*, 331(3), 690-702. <https://doi.org/10.1016/j.jhydrol.2006.06.012>
